# Virulence Factors Associated with *Enterococcus Faecalis* Infective Endocarditis: A Mini Review

**DOI:** 10.2174/1874285801711010001

**Published:** 2017-03-31

**Authors:** Kristian T. Madsen, Marianne N. Skov, Sabine Gill, Michael Kemp

**Affiliations:** 1 Dept. of Clinical Microbiology, Odense University Hospital and Clinical Department, University of Southern Denmark, Denmark; 2 Dept. of Cardiology, Odense University Hospital, Odense, Denmark

**Keywords:** Biofilms, Enterococcus, *Enterococcus faecalis*, Endocarditis, Review, Virulence Factors

## Abstract

**Introduction:**

The enterococci are accountable for up to 20% of all cases of infective endocarditis, with *Enterococcus faecalis* being the primary causative isolate. Infective endocarditis is a life-threatening infection of the endocardium that results in the formation of vegetations. Based on a literature review, this paper provides an overview of the virulence factors associated with *E. faecalis* infective endocarditis. Furthermore, it reports the effects of active or passive immunization against some of these involved factors.

**Individual virulence factors:**

Nine virulence factors have in particular been associated with *E. faecalis* infective endocarditis. Absence of these factors entailed attenuation of strains in both mixed- and mono-bacterial infection endocarditis models as well as in *in vitro* and *ex vivo* assays when compared to their virulence factor expressing parental strains.

**Pathogenesis:**

The virulence factors promote a broad spectrum of events that together allow for disease development and progression. The infection is initiated through bacterial binding to ligands present at the site of infection after which the colonization can be accelerated through inter-bacterial attachment and modulation of the host immune response. The formation and growth of the vegetation provide protection and promote growth. Controlled degeneration of the vegetation appears to increase the likelihood of embolization and dissemination, without exposing protected bacteria.

**Prophylactic immunization:**

In most cases, active and passive immunization against associated virulence factors provided partial protection.

**Future prospects:**

There is a need for further evaluation of the known virulence factors. Immunization against two or more virulence factors might be an effective prophylactic tool.

## INTRODUCTION

Some of the first microbial colonizers of the infant gastrointestinal tract are the enterococci. They were once regarded as commensal intestinal organisms with minimal clinical importance, but since the mid to late 1970s, they have emerged as opportunistic pathogens capable of causing infective endocarditis (IE), bacteremia, intraabdominal infections, wound infections, and urinary tract infections, among others, especially in the hospital setting [1-3]. Enterococci are accountable for up to 20% of all cases of bacterial IE [4-7], with *E. faecalis* being the causative isolate in >90% of the cases [3, 8].

IE is an infection of the endocardium that results in the formation of lesions termed septic vegetations, which are clotted masses of platelets, fibrin, bacteria, and immune cells, that without effective therapy may lead to valvular destruction, congestive heart failure, and death [4, 9]. *E. faecalis* IE is a severe infection that still has an in hospital mortality of 15 to 20% and a one year mortality of approximately 25 to 30% [8, 10].

The course of *E. faecalis* IE is initiated through bacteremia, caused by events such as surgical procedures, ascending urinary tract infections or intestinal bacterial translocation. This is followed by attachment of the bacteria either directly to the inner linings of the endothelium, to a valvular prosthesis or to a sterile vegetation, consisting of fibrin and platelets, formed due to prior valvular damage. Once initial attachment is achieved, the bacteria become encased in a matrix of platelets and fibrin that protects them from the recruited immune cells leading to the creation of a septic vegetation. The infective endocarditis vegetation is a biofilm process on the heart valves, which provides a protective barrier as well as a favorable growth environment, in which the bacteria are more resistant to antibiotics and immune mediated killing. Furthermore, the vegetation also potentially allows for an increased horizontal gene transfer as well as providing a vessel for dissemination of bacteria to other organs, when pieces of the vegetation break off [1, 4, 5, 9].

This mini review comprises an outline of the current knowledge regarding the virulence factors associated with *E. faecalis* IE and their individual contribution to the pathogenesis. Furthermore, it reports findings regarding the ability of active or passive immunization against involved virulence factors to provide protection against the disease.

Data was obtained through a PubMed literature search and a flowchart of the conducted literature search, including the inclusion and exclusion criteria, is shown in Fig. (**1**). The search was not restricted to a certain timeframe, but the exclusion criteria seek to eliminate older redundant studies.

The review of the included literature revealed nine virulence factors, involved in the *E. faecalis* IE pathogenesis. In addition to the papers identified from the literature search, one original paper [11] cited by one of the identified papers and two papers describing clinical background information [8, 10] were included in this article.

In the following section, the results, pertaining to each identified virulence factor are presented together with a brief introduction.

## INDIVIDUAL VIRULENCE FACTORS

A chronological overview of the obtained results and their corresponding p-values is available in Table **1**.

### Aggregation Substance (AS)

AS is a large pheromone inducible surface protein of *E. faecalis*, known to be involved in bacterial aggregation through binding to enterococcal binding substance (EBS). So far, three different AS proteins (Asa1, Asc10 and Asp1) have been identified, each encoded by their own pheromone-responsive conjugative plasmid (pAD1, pCF10 and pPD1, respectively). Together they comprise a family of surface adhesins that are almost identical throughout most of the protein, the exception being a small variable segment with a shared identity of about 30 to 40% [4, 12].

Asc10 appears to be the most extensively studied AS protein in relation to *E. faecalis* IE, with four included articles [4, 5, 12, 13] investigating the role of this particular protein, compared to one article [14] investigating Asa1 and no identified articles investigating Asp1.

Asc10 is encoded by the *prgB* gene of pCF10 and several functional domains of the protein have been described, amongst these, an N-terminal and a central aggregation domain as well as an N- and C-terminal Arg-Gly-Asp (RGD) motif [4].

In 1998 Schlievert, *et al.* [13], used a rabbit model of endocarditis to investigate the role of Asc10 and EBS in *E. faecalis* IE. They found that not only did the combination of Asc10 and EBS expression increase lethality, but it also prevented pericardial inflammation [13]. Later, in 2008 Chuang, *et al.* [4], investigated the individual roles of different functional domains of Asc10 in *E. faecalis* IE. Using a rabbit model of endocarditis, they found that a strain with mutations to both RGD motifs exhibited virulence that was essentially equivalent to that of a strain carrying the *prgB* null allele. Furthermore, mutations to either RGD motif alone, revealed that the N-terminal RGD motif seems to be more important for virulence than the C-terminal RGD motif. Disruption of either aggregation domain resulted in strains that were less virulent than the wild-type strain, but more virulent than the double RGD mutant [4].

In a subsequent study, Chuang-Smith, *et al.*, 2010 [5] used an *ex vivo* porcine heart valve model to evaluate the role of Asc10 in bacterial adherence and biofilm formation at early time points. They found that strains, regardless of Asc10 expression, were able to attach to and initiate biofilm development on the heart valves with similar colonization for the first two hours. However, after four hours, the Asc10^-^ strain was found to colonize the valves at only 40% of the level of the Asc10^+^ strain. Furthermore, they observed that the micro colonies of the Asc10^+^ strain were large, containing hundreds of bacteria and abundant extracellular matrix (ECM), whereas the Asc10^-^ strain produced smaller micro colonies, with less ECM. They also assessed the contribution of the aforementioned functional domains of Asc10 with regards to initial adherence and biofilm formation, and found that several of the mutations had less severe effects in their *ex vivo* valve model, compared to the *in vivo* endocarditis model used by Chuang, *et al.*, 2008 [4, 5]

### Hemolysin

The enterococcal hemolysin is encoded on the pheromone-responsive plasmid pAD1 [14], the same plasmid which also encodes the AS protein Asa1 [4]. Chow, *et al.*, 1993 [14] used a rabbit model of endocarditis to evaluate strains that expressed hemolysin and/or Asa1. They observed a 55% mortality in the hemolysin^+^ Asa1^+^ group, a 15% mortality in the hemolysin^–^ Asa1^+^ group and a 0% mortality in the hemolysin^+^ Asa1^-^ group. Furthermore, they found that the Asa1^+^ strains produced vegetations with weights varying from 181-209 mg, whereas the Asa1^-^ strains produced vegetations with weights varying from 81-92 mg [14].

### Cell-Wall Glycolipids

Studies have shown that mutant *E. faecalis* strains with impaired or eliminated glycolipid synthesis, exhibit impaired biofilm formation, a process known to play a crucial role in IE. In *E. faecalis*, two glucosyltransferases, termed biofilm-associated glycolipid synthesis A and B (bgsA and bgsB, respectively), are responsible for the biosynthesis of the two main cell wall glycolipids, diglycosyl-diacylglycerol and its precursor monoglycosyl-diacylglycerol. BgsA deficiency has been shown to lead to a loss of diglycosyl-diacylglycerol in the cell membrane and to an accumulation of monoglycosyl-diacylglycerol, whilst bgsB deficiency leads to a complete loss of cell membrane glycolipids [15].

In 2014 Haller, *et al.* [15], assessed the importance of glycolipids in *E. faecalis* IE. Using a rat model of endocarditis, they compared mutant strains, deficient for either bgsA or bgsB, with a parental wild-type strain. They found that both of the mutant strains were attenuated, compared to the wild-type strain, producing vegetations that weighed less and contained significantly less CFU per milliliter and per gram. The bgsB mutant exhibited the highest level of attenuation [15].

### General Stress Protein 24 (gls24)

Gls24 is a protein, synthesized in *E. faecalis* during stress conditions such as glucose starvation, and exposure to cadmium chloride or bile salts [7]. In 2005 Nannini, *et al.* [7], compared a gls24 disruption mutant with its parental wild-type strain (OG1RF). Using a mixed-infection rat model of endocarditis, they found that OG1RF outnumbered the gls24^-^ strain, constituting 77% and 84% of the mean percentage of the total number of bacteria recovered from vegetations at 72 and 120 hours, respectively. The initial injection mixtures used for the experiments contained 36% and 43% OG1RF, respectively. The researchers also calculated the mean virulence index of the gls24^-^ strain relative to OG1RF and found it to be 0.18 and 0.09 at 72 and 120 hours, respectively [7].

### Endocarditis and Biofilm-Associated Pili (Ebp) of E. Faecalis

Pili and fimbriae are fibrous protein organelles, anchored to the surface of the bacterium, that interact with the external environment. They are known to be used by some bacteria to accomplish adhesion to host cells [1, 9]. In 1981 fimbriae-like structures were reported on the surface of *E. faecalis*, and today it is known that their assembly is dependent on a three-gene EbpABC locus and an associated sortase gene, *srtC*. High titers of antibodies against the EbpABC proteins have been found to be present in the sera of patients suffering from *E. faecalis* IE, thus indicating that they are readily expressed and recognized by the host immune system during infection [9].

In 2006 Nallapareddy, *et al.* [9], assessed the role of the EbpA-srtC gene cluster in biofilm formation, initial attachment and IE. They found that mutations within the EbpA–srtC gene cluster markedly reduced the ability of the strains to form biofilms. Furthermore, a two hours *in vitro* attachment assay revealed significant differences in initial attachment, with the wild-type parental strain (OG1RF) exhibiting a higher number of adherent cells per microscopic field, than any of the mutant strains. Lastly, the researchers used a mixed-infection rat model of endocarditis, in which they injected the rats with an equal mixture of a nonpiliated EbpA mutant and OG1RF. They found, that the mean percentage of the EbpA mutant in the total CFU of bacteria recovered from vegetations after 24 hours was 26.1% and calculated the mean virulence index of the EbpA mutant relative to OG1RF to be 0.06 [9].

### Gelatinase (GelE)

GelE is an extracellular zinc-metalloprotease that is thought to contribute to virulence through degradation of host substrates such as collagen, fibrinogen, fibrin, and complement components C3 and C3a among others. It is one of two proteases of *E. faecalis*, the other one being serine protease (SprE) [6].

In 2010 Thurlow, *et al.* [6], sought to elucidate the relative contribution of each protease to *E. faecalis* IE by comparing the vancomycin-resistant *E. faecalis* V583 with three mutant strains that lacked either GelE and/or SprE. They also assessed if any of the two proteases possessed proteolytic activity towards complement component C5a. Using a rabbit endocarditis model, they found no significant differences in bacterial burdens on the heart valves for any of the strains tested. However, an assessment of the mean CFU per gram of heart tissue, revealed a 14- and 7.2-fold reduction for the GelE^-^ SprE^+^ and GelE^-^ SprE^-^ strains when compared to V583. Conversely, they observed a 6.5-fold increase in the mean CFU per gram of heart tissue for the GelE^+^ SprE^-^ strain when compared to the GelE^-^ SprE^+^ strain. Lastly, the researchers found that GelE is capable of hydrolyzing C5a [6].

### Adhesin to Collagen of E. Faecalis (Ace)

Ace is a cell-wall anchored adhesin, shown to be antigenic during human *E. faecalis* IE as well as playing a vital role in the *in vitro* adherence of *E. faecalis* to immobilized collagen. The heart valves, the aortic tissue, as well as sterile vegetations are all known to be collagen-containing sites [3].

In 2010 Singh, *et al.* [3], assessed the role of Ace in *E. faecalis* IE, using an *in vitro* adherence assay as well as mixed- and mono-infection rat endocarditis models. They tested four different strains; OG1RF (parental wild-type strain), OG1RFΔace (Ace deletion mutant), pAT392::ace (Ace deletion mutant containing a shuttle vector with a cloned Ace gene) and pAT392 (Ace deletion mutant, containing an empty shuttle vector). The percentages of Ace-expressing cells in cultures of OG1RF, OG1RFΔace, pAT392 and pAT392::ace were >70%, <5%, <5% and >90%, respectively. In the adherence assay they found that both of the Ace^-^ strains (OG1RFΔace and pAT392) were highly attenuated, compared to OG1RF, and even more so compared to pAT392::ace, in adherence to collagen type I and IV. To assess the importance of Ace *in vivo*, the researchers used a mixed-infection rat model of endocarditis, in which rats were injected with a 1:1 mixture of OG1RF and OG1RFΔace. After 72 hours, they recovered the bacteria from the valves and found the mean percentage of OG1RFΔace in the total CFU to be 18.5%, which corresponds to a mean virulence index of OG1RFΔace relative to OG1RF of 0.077. Lastly, they wanted to assess the role of Ace in the early attachment stage of valve colonization. To do so, they compared the results of two independent mono-infection models, in which rats were challenged with either pAT392 or pAT392::ace. They found that after four hours, valve vegetations of rats infected with pAT392::ace contained 1.4±0.6 log_10_ more CFU/geometric mean, than valve vegetations of rats infected with pAT392 [3].

### Enterococcal Fibronectin-Binding Protein A (EfbA)

EfbA is an adhesin, localized on the outer surface of *E. faecalis* that confers adhesion to immobilized fibronectin. Fibronectin is a glycoprotein that, among other places, can be found in the ECM of the cardiac endothelium. It is exposed if the cardiac tissue gets damaged, whereupon it binds fibrin and platelets, thus contributing to the formation of a sterile vegetation [2].

In 2015 Singh, *et al.* [2], sought to assess the role of EfbA in *E. faecalis* IE. Using a microtiter plate-based assay, they demonstrated that recombinant EfbA (rEfbA) exhibits a concentration-dependent binding to immobilized human fibronectin as well as high affinity to collagen I and V. Furthermore, they showed that an EfbA deletion mutant (TX5707) showed a 44% decrease in whole cell binding to immobilized fibronectin compared to the wild-type parental strain (OG1RF). A derivative strain, in which the EfbA gene had been restored in its original chromosomal location (TX5708), showed whole cell binding to immobilized fibronectin similar to that of OG1RF. When examined for their ability to form biofilms, TX5707 was found to produce biofilms with a 15% reduced density compared to OG1RF and TX5708. To assess the contribution of EfbA to *E. faecalis* IE, the researchers used a mixed-infection rat model of endocarditis. First, they injected rats with a 1:1 mixture of OG1RF and TX5707 and after 48 hours, the mean percentage of TX5707 in the total CFU of bacteria recovered from the valves was found to be 25%. Next, they injected the rats with a mixture of 59% TX5707 and 41% TX5708 and after 48 hours, the mean percentage of TX5707 in the total CFU of bacteria recovered from the valves was found to be approximately 37% [2].

### Eep

Eep is a membrane metalloprotease, that is known for its role in the proteolytic processing of sex pheromone peptides that, once matured, induce conjugation in a class of large pheromone-responsive conjugative plasmids, such as pCF10 and pAD1 [16].

Frank, *et al.*, 2011 [16] used a rabbit model of endocarditis in which they tested a plasmid-free version of OG1RF and an Eep mutant strain (OG1RFΔeep) to assess the role of Eep in *E. faecalis* IE. They found that OG1RFΔeep exhibited a 4-log_10_ decrease in valve bacterial load compared to OG1RF. Furthermore, they observed that OG1RFΔeep produced small vegetations with a mean weight of 8.8 mg, whilst OG1RF produced large vegetations with a mean weight of 47.6 mg. The researchers also assessed a strain that had been complemented with the wild-type Eep locus *in trans* on a plasmid. They found that the complemented strain produced vegetations with a mean weight of 32.1 mg, despite the fact that only 2% of the cells retained the plasmid at the end of the experiment [16].

## PATHOGENESIS

An overview of the assessed contribution of each virulence factor, to different steps of the disease process is available in Table **2**.

### Early Stages of Infection and Colonization

The first step of IE, once bacteria have entered the bloodstream, is the initial attachment to and colonization of the inner linings of the heart, the valve prosthesis or the sterile vegetation.

Asc10, Ace and EfbA were all found to mediate binding to substances that are either exposed once the endocardium is damaged or present in the following sterile vegetation [2-4]. The role of Asc10 in the initial attachment process is presumably conferred through binding to fibrin, since Chuang, *et al.*, 2008 [4] could not identify any other ligands for Asc10 mediated binding [4]. It does however, seem that the contribution of Asc10 to the initial attachment process is redundant, compared to that of other factors. This was proposed by Chuang-Smith, *et al.*, 2010 [5], who observed that for the first two hours all test strains, regardless of Asc10 expression, could adhere to and initiate biofilm formation on the valves at equal rates. The differences they observed in colonization after four hours, indicates that the major contribution of Asc10 to the early stages of infection is through acceleration of the adherence of microbes, once initial attachment has been achieved. Since the AS proteins are known to be involved in the process of bacterial aggregation, through binding to EBS [13], it could be hypothesized that this acceleration is due to Asc10 allowing for the adherence of planktonic cells as well as blood formed bacterial aggregates to already attached bacteria [5]. The AS proteins are closely related and thus the Asc10 results might provide an explanation for why Chow, *et al.*, 1993 [14] observed that hemolysin and Asa1 expression had a synergistic effect on rabbit mortality. If so, it might be that Asa1 increased the number of adherent bacteria, hereby not only increasing the size of the vegetation but also allowing for the manifestation of hemolysin´s toxic effects, which in turn increased rabbit mortality.

The importance of Ace in the initial attachment process was highlighted by the results of the 4 hours endocarditis experiment conducted by Singh, *et al.*, 2010 [3], in which an Ace mutant was highly attenuated compared to an Ace expressing strain. The results obtained from their *in vitro* adherence assay suggests, that the mechanism behind Ace mediated attachment is likely through binding to either collagen I or IV [3].

EfbA was found to mediate binding to fibronectin, collagen I and collagen V, and thus the observed attenuation of an EfbA mutant in an endocarditis model as observed by Singh, *et al.*, 2015 [2] could be explained by a reduced EfbA mediated binding to these substances. The data available for EfbA are however insufficient in order to, with approximate certainty, exclude other possible reasons for the observed differences, such as increased vulnerability of the mutant to the host immune system among others.

Two other factors, Eep and the Ebp pili, also seem to be important during the early stages of infection, but unlike Asc10, Ace and EfbA, the data obtained do not reveal any putative ligands for adherence.

Eep is known to be involved in the maturation of pheromone responsive plasmids that encode the AS proteins among others. However, since Frank, *et al.*, 2011 [16] used a plasmid-free version of OG1RF in their experiments, their data describe functions of Eep, that does not directly involve its effects on pheromone-inducible conjugative plasmids. Their results, showing that a strain complemented with Eep on a plasmid was able to produce vegetations with a mean weight close to that of the wild-type strain, despite only 2% of the cells retaining the plasmid at the end of the experiment, suggests that Eep is important during the early stages of infection [16]. The specific mechanisms behind the contribution of Eep to the *E. faecalis* IE pathogenesis remain unknown and further studies are needed to elucidate this, but it seems clear that Eep plays a role that goes beyond the maturation of pheromone-responsive plasmids.

Likewise, the observations made by Nallapareddy, *et al.*, 2006 [9] showing attenuation of non-piliated strains in both an initial attachment assay as well as in a mixed endocarditis model, highlights the importance of the Ebp pili in the early stages of infection, but without giving clues to the specific mechanisms behind this process.

### Biofilm Formation and Maintenance

The IE vegetation is a biofilm process on the heart valves [9]. A biofilm is essentially a microbial community/multicellular microcolony, arisen from the adherence of planktonic organisms, which then typically synthesize an ECM, which helps to stabilize them physically [1, 5].

Three identified virulence factors seem to be involved in the formation of biofilms during *E. faecalis* IE. Asc10 was observed to contribute to the establishment of large microcolonies containing abundant ECM [5], a non-piliated EbpA mutant was found to produce biofilms with a density similar to that of a non-biofilm producer [9] and an EfbA mutant produced biofilms with a 15% reduced density compared to its parental strain [2].

The formation and accumulation of the vegetation is known to change both the growth as well as the physiological state of the bacteria, which in turn impedes the effectiveness of antibiotics and the host immune system and it is thus advantageous for the bacteria [5]. Interestingly, it appears that controlled degradation of the vegetation can make it even more advantageous. The protease GelE seems to be involved in this process through degradation of the fibrin layer. The GelE-mediated degradation appears to be thinning the biofilm so that it becomes unstable enough for pieces to break off and embolize, without thinning it so much that encased bacteria become exposed and vulnerable. This hypothesis arises from Thurlow, *et al.*, 2010 [6], who found that a mutation to GelE did not cause a significant reduction in bacterial burden on the valves, but markedly decreased the total number of CFU per gram of heart tissue, thus indicating decreased bacterial dissemination. Furthermore, through histological examination, they found that the matrix layer of the aortic vegetations infected by GelE mutants was 10-fold thicker compared to the layer of vegetations infected by the parental strain [6].

### Immune Modulation

The data obtained from the included studies reveal that both Asc10 and GelE, might be involved in the process of immune modulation. The first indications of Asc10´s role in immune modulation came from results obtained in 1998 by Schlievert, *et al.* [13], who found that Asc10 prevented pericardial inflammation and from results obtained in 1999 by Rakita, *et al.* [11], who found that Asc10 expression made *E. faecalis* resistant to killing following ingestion by PMNs. In 2010 Chuang-Smith, *et al.* [5], observed that mutations to the RGD motifs of Asc10 had a much less severe impact on virulence in their *ex vivo* model compared to the *in vivo* results obtained by Chuang, *et al.*, 2008 [4]. Since there is no host immune system present in the *ex vivo* model, it is possible, that the observed differences are due to involvement of the RGD motifs in immune evasion.

GelE also seems to be involved in immune modulation, as Thurlow, *et al.*, 2010 [6] found that in addition to being able to cleave C3a it can also cleave the 100 times more potent complement protein C5a, resulting in a decreased heterophil migration to the infected tissue sites *in vivo* [6]. Despite this, they observed no significant differences in the numbers of bacteria colonizing the aortic valve regardless of GelE expression, indicating that the cleavage of C5a might play a minor role in IE, perhaps because the vegetation provides protection.

### Factors With Uncertain Contribution

The existing data regarding gls24 reveal that it is important for virulence in *E. faecalis* IE, but it does not offer an exact explanation to its specific contribution.

The cell-wall glycolipids of *E. faecalis* are known to take part in biofilm formation [15], thus the reduced virulence of strains with altered or abolished glycolipid synthesis observed by Haller, *et al.*, 2014 [15], could be due to reduced biofilm formation capabilities of these strains, but more data are needed in order to elucidate this.

## PROPHYLACTIC IMMUNIZATION

The current management of *E. faecalis* IE, revolves around antimicrobial therapy in the form of administration of a combination of antibiotics often alongside with surgery [3]. Strains of *E. faecalis* that are resistant towards the most commonly used antibiotics are complicating the treatment and has created the need for alternative ways of dealing with the infection [2, 3]. One possible course of action is prophylactic immunization.

Experiments assessing the ability of active and/or passive immunization against the virulence factors AS, Ace, Ebp pili and EfbA, to prevent or reduce the severity of the infection in animal models have been conducted by Schlievert, *et al.*, 2010 [12], Singh, *et al.*, 2010 [3], Pinkston, *et al.*, 2014 [1] and Singh, *et al.*, 2015 [2], respectively.

Their collective results revealed that immunization with IgG antibodies against AS enhanced vegetation formation and caused extensive lung congestion in test animals. Conversely, immunization with IgG antigen binding fragments (Fabs) against AS, reduced both the mean weight of vegetations from 40 mg to 10 mg as well as the number of *E. faecalis* in vegetations by more than two log CFUs [12]. Both active immunization with recombinant Ace (rAce) as well as passive immunization with anti-rAce antibodies made test animals less susceptible to infection and reduced the number of bacteria in vegetations [3]. Passive immunization with an anti-pilin monoclonal antibody (MAb), termed MAb 69, which exhibits an *in vitro* dose-dependent inhibition on biofilm formation, resulted in 3/10 rats developing *E. faecalis* IE compared to 9/10 rats in the control group [1]. Vaccination with rEfbA, reduced both the percentage of rats developing IE from 95% to 60% and the mean log_10_ CFU bacteria in the vegetations from 4.8 to 2.1 [2].

An overview of the obtained immunization results and their corresponding p-values is available in Table **3**.

These results indicate that it is possible to achieve partial protection against *E. faecalis* IE, in animal models, through active and passive immunization against factors that are putatively involved in the initial attachment process. However, it must be taken into account that immunization against an individual virulence factor provides the host with a factor-specific immune response that might only be effective against strains expressing that particular factor. Thus, targeting a frequently expressed virulence factor could prove effective against many genetically different isolates.

Studies have shown that 90% of patients with prior *E. faecalis* IE have antibodies against Ace in their sera. The Ace gene is also ubiquitously present in *E. faecalis* isolates and its amino acid sequence is highly conserved [3], indicating that immunization against Ace might be effective in most cases. Comparatively, studies have found that <45% of *E. faecalis* isolates produce AS [3]. Furthermore, as immunization against Ace as well as any of the other tested factors, did not offer complete protection against *E. faecalis* IE, it could be necessary to target multiple factors in order to achieve a protection that is more robust. Both the Ace gene and the Ebp locus are ubiquitously present in *E. faecalis* isolates [3, 9], thus they could be of interest in this regard.

Lastly, it must be taken into account, that the people who are at the greatest risk of getting IE, are those who are either immunocompromised due to chemotherapy or weakened by age, underlying diseases or surgical treatments [1]. This patient group will most likely not be able to mount effective immune responses and thus active immunization might be a less viable option for this high-risk group, unless long lasting protection can be achieved prior to disease or aging.

## FUTURE PROSPECTS

The review of the included articles has revealed that there is still a need for further studies evaluating the known virulence factors associated with *E. faecalis* IE. Furthermore, this study has not addressed the activation and transfer of the genes encoding the virulence factors, nor has it addressed how *E. faecalis* manages to enter the bloodstream. Future reviews elucidating these areas might provide even further insight into the understanding of the disease, as well as new possible ways to prevent it. Lastly, the emergence of multi-resistant *E. faecalis* strains, complicating the treatment, means that it is important to search for and identify new treatment strategies. Immunization against two or more virulence factors might prove to be an effective prophylactic tool, but further studies are needed to elucidate this. For treatment of patients suffering from *E. faecalis* IE, the best course of action may include attacking the vegetation, in order to expose the bacteria and make them vulnerable to antibiotics and the host immune system, but bearing the results from the GelE experiments in mind, such an approach could also end up increasing the dissemination of bacteria.

## Figures and Tables

**Fig. (1):**
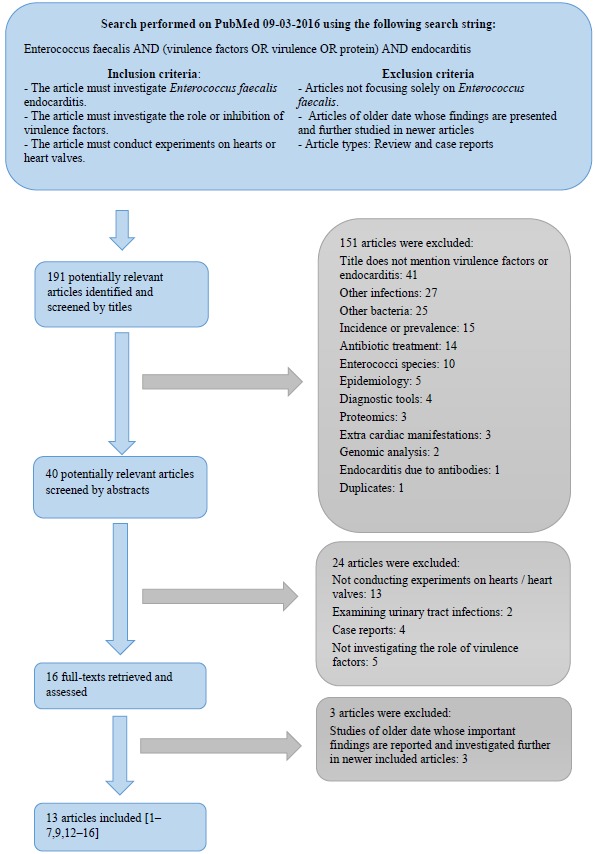
Flowchart of the literature search.

**Table 1:** Infection and biofilm results and corresponding p-values.

**Article**	**Studying**	**Results**	**P value**
Schlievert, *et al.*, 1998 [13]	Asc10 and EBS	The combination of Asc10 and EBS increase lethality.	0.0002
Chuang, *et al.*, 2008 [4]	Asc10	A double RGD mutant exhibited virulence equivalent to that of a strain carrying the *prgB* null allele.	≤ 0.01
		The N-terminal RGD motif is more important for virulence than the C-terminal RGD motif.	≤ 0.01
		Strains with mutations to the aggregation domains are more virulent than double RGD motif mutants.	≤ 0.01
Chuang-Smith, *et al.*, 2010 [5]	Asc10	After 4 hours the Asc10^-^ strain colonized heart valves at 40% the level of the Asc10^+^ strain.	n/a*
		Several mutations had less severe impact on virulence in the *ex vivo* model.	≤ 0.02
Chow, *et al.*, 1993 [14]	Asa1 and hemolysin	Strains expressing Asa1 and hemolysin exhibited the highest rabbit mortality.	< 0.01
		Asa1^+^ strains produces vegetations weighing more than those produced by Asa1^-^ strains.	< 0.01
Haller, *et al.*, 2014 [15]	Cell-wall glycolipids	bgsA mutants produced vegetations that weighed less than the wild type.	< 0.35
		bgsB mutants produced vegetations that weighed less than the wild type.	< 0.05
		bgsA and bgsB mutants produced vegetations that contained less CFU/gram than the wild type.	< 0.0159
Nannini, *et al.*, 2005 [7]	Gls24	A Gls24 mutant was outnumbered by its parental strain at both time points in an endocarditis model.	< 0.001
Nallapareddy, *et al.*, 2006 [9]	Ebp pili	Mutant strains exhibited markedly reduced biofilm formation.	< 0.001
		Mutant strains were attenuated compared to the parental strain in a 2 hours in vitro attachment assay.	< 0.001
		An EbpA mutant was outnumbered by its parental strain in an endocarditis model.	0.0069
Thurlow, *et al.*, 2010 [6]	GelE	Mutations to GelE significantly decreased the mean CFU per gram of heart tissue	< 0.05
		Mutations to GelE significantly increases the matrix layer	< 0.05
Singh, *et al.*, 2010 [3]	Ace	Ace^-^ strains were highly attenuated in adherence to collagen type I and IV compared to Ace^+^ strains	< 0.0001
		OG1RFΔace was outnumbered by OG1RF in a mixed endocarditis model	< 0.0001
		At 4 hours pAT392 produced vegetations with less CFU/geometric mean than pAT392::ace	0.0417
Singh, *et al.*, 2015 [2]	EfbA	TX5707 mutant showed a 44% decrease in whole cell binding to immobilized fibronectin	< 0.0001
		TX5707 produced biofilms with a 15% reduced density compared to OG1RF and TX5708	< 0.0001
		At 48 hours TX5707 was outnumbered by OG1RF in a mixed endocarditis model	< 0.0006
		At 48 hours TX5707 was outnumbered by TX5708 om a mixed endocarditis model	< 0.0086
Frank, *et al.*, 2011 [16]	Eep	OG1RFΔeep exhibited a 4-log_10_ decrease in valve bacterial load compared to OG1RF	0.003
		OG1RFΔeep produced smaller vegetations (mean 8.8 mg) than OG1RF (mean 47.6 mg)	0.0065
		A complemented strain produced larger vegetations (mean 32.1) than OG1RFΔeep (mean 8.8 mg)	0.027
* n/a indicates that the corresponding p-value is not presented in the article.			

**Table 2:** Assessed contribution of each virulence factor.

**Virulence factor**	**Initial attachment**	**Bacterial aggregation**	**Biofilm formation**	**Biofilm degradation**	**Immune modulation**	Contribution to *E. faecalis* IE*
Ace	+++	-	-	-	-	+++
Asc10	+	+++	++	-	++	+++
Cell-wall glycolipids	-	-	+	-	-	++
Ebp pili	+++	-	+++	-	-	+++
Eep	++	-	-	-	-	++
EfbA	++	-	+	-	-	++
GelE	-	-	-	+++	+	++
Gls24	-	-	-	-	-	++
Hemolysin	-	-	-	-	-	+
The virulence factors have been assigned a score of either +, ++, +++ or -.						
- indicates that the reviewed data does not support involvement of the virulence factor.						
+ indicates that the data suggests little involvement, ++ intermediate involvement and +++ high involvement.						
* The relative contribution of each virulence factor to the disease as compared to other involved factors.						

**Table 3:** Immunization results and corresponding p-values.

**Article**	**Studying**	**Results**	**P value**
Schlievert, *et al.*, 2010 [12]	IgGs and IgG Fabs against AS	Immunization with IgGs against AS increased vegetation formation when exposed to Asc^+^ strains	0.002
		Immunization with IgG Fabs reduces vegetation formation when challenged with Asc^+^ strains	0.05
Singh, *et al.*, 2010 [3]	rAce and anti-rAce antibodies	Active immunization with rAce reduced the number of infected rats	0.0001
		Active immunization with rAce reduced the number of bacteria in the vegetations	0.0004
		Passive immunization with anti-rAce antibodies reduced the number of bacteria in the vegetations	0.0231
Pinkston, *et al.*, 2014 [1]	MAb 69	3/10 rats given MAb 69 developed endocarditis compared to 9/10 in the control group	0.0123
Singh, *et al.*, 2015 [2]	rEfbA	rEfbA provided protection and reduced the total CFU bacteria recovered from vegetations	0.008

## ABBREVIATIONS

Ace: = Adhesin to collagen of *E. faecalis*
AS: = Aggregation substance
bgsA: = Biofilm-associated glycolipid synthesis A
bgsB: = Biofilm-associated glycolipid synthesis B
Ebp: = Endocarditis and biofilm-associated pili
EBS: = Enterococcal binding substance
ECM: = Extracellular matrix
EfbA: = Enterococcal fibronectin-binding protein A
Fab: = Fragment antigen binding
GelE: = Gelatinase
gls24: = General stress protein 24
IE: = Infective endocarditis
MAb: = Monoclonal antibodies
PMN: = Polymorphonuclear leukocyte
rAce: = Recombinant Ace
rEfbA: = Recombinant Efba
RGD: = Arg-Gly-Asp
SprE: = Serine protease
